# Mutation Study of Malaysian Patients with Ornithine Transcarbamylase Deficiency: Clinical, Molecular, and Bioinformatics Analyses of Two Novel Missense Mutations of the* OTC* Gene

**DOI:** 10.1155/2018/4320831

**Published:** 2018-08-05

**Authors:** Ernie Zuraida Ali, Yuslina Zakaria, Mohd Amran Mohd Radzi, Lock Hock Ngu, Siti Azma Jusoh

**Affiliations:** ^1^Molecular Diagnostics and Protein Unit, Specialized Diagnostics Centre, Institute for Medical Research (IMR), Jalan Pahang, 50588 Kuala Lumpur, Malaysia; ^2^Faculty of Pharmacy, Universiti Teknologi MARA (UiTM), Puncak Alam Campus, 42300 Bandar Puncak Alam, Selangor, Malaysia; ^3^Department of Electrical and Electronic Engineering, Universiti Putra Malaysia (UPM), 43400 Serdang, Selangor, Malaysia; ^4^Genetics Department, Kuala Lumpur Hospital, Jalan Pahang, 50586 Kuala Lumpur, Malaysia

## Abstract

Ornithine transcarbamylase deficiency (OTCD), an X-linked disorder that results from mutations in the* OTC* gene, causes hyperammonemia and leads to various clinical manifestations. Mutations occurring close to the catalytic site of OTCase can cause severe OTCD phenotypes compared with those caused by mutations occurring on the surface of this protein. In this study, we report two novel* OTC* missense mutations, Q171H and N199H, found in Malaysian patients. Q171H and N199H caused neonatal onset OTCD in a male and late OTCD in a female, respectively. In silico predictions and molecular docking were performed to examine the effect of these novel mutations, and the results were compared with other 30 known* OTC* mutations. In silico servers predicted that Q171H and N199H, as well as 30 known missense mutations, led to the development of OTCD. Docking analysis indicated that N-(phosphonoacetyl)-L-ornithine (PALO) was bound to the catalytic site of OTCase mutant structure with minimal conformational changes. However, the mutations disrupted interatomic interactions in the catalytic site. Therefore, depending on the severity of disruption occurring at the catalytic site, the mutation may affect the efficiency of mechanism and functions of OTCase.

## 1. Introduction

Ornithine transcarbamylase (OTCase; EC 2.1.3.3) catalyzes the formation of citrulline from ornithine (ORN) and carbamoyl phosphate (CP) in the liver and small intestine [1–3]. Mutations in the* OTC* gene cause OTC deficiency (OTCD; OMIM 311250), an X-linked recessive disorder. This is the most frequent inborn defect of the urea cycle, leading to the accumulation of ammonia. Hemizygous males with complete OTCD exhibit acute hyperammonemia in the neonatal period, whereas heterozygous females and hemizygous males with partial defects show various degrees of clinical symptoms at a later age [4]. The mainstays of treating acute hyperammonemia involve rapid lowering of the plasma ammonia level via pharmacological removal of ammonia with ammonia scavengers. This allows the excretion of excess nitrogen via alternative pathways or, if necessary, extracorporeal detoxification of ammonia with renal replacement therapy and reversal of catabolism. Treatment can reduce the risk of neurological damage. Prolonged treatment is usually aimed at hindering the hyperammonemic episodes and encouraging growth and development. In severe cases, a liver transplant is normally performed by the age of 6 months to avoid continuous hyperammonemic crises and neurodevelopmental deterioration. Liver transplant is also typically considered for females and males with partial OTC deficiency who have frequent hyperammonemic episodes [5–9].

The human* OTC* gene, located on the short arm of X chromosome (Xp21.1), is comprised of 10 exons and nine introns and is expressed only in the liver and intestinal mucosa [10]. The gene encodes a precursor of 354 amino acids and matures when the 32 residues at its N-terminal region are cleaved into a 322-residue polypeptide, which later forms a homotrimer [11]. Each monomer has a catalytic site for CP and ORN binding, located at the base of the cleft between the two lobes and at the edge of the cleft, respectively. There are, currently, three human OTCase crystal structures: (1) OTCase crystallized with PALO (a bisubstrate analogue) (PDB ID: 1OTH) [12]; (2) OTCase crystallized with CP and L-norvaline (inhibitor/substrate analogue of ornithine) (PDB ID: 1C9Y) [13]; and (3) OTCase crystallized with CP (PDB ID: 1EP9 and 1FVO) [14]. These structures reveal specific interactions between OTCase and the substrates.

To date, more than 400 disease-causing mutations of the* OTC *gene have been compiled [3]. Pathogenic variants of the* OTC* gene include nonsense, frameshift, and missense variants, as well as small deletions or insertions, splice site mutations, large deletions, complex rearrangements, and regulatory mutations. The most commonly reported pathogenic variants are missense variants, which are distributed to all protein regions. The changes in amino acids, resulting from these single nucleotide mutations, are dominantly clustered in the regions near the binding pockets of the CP and ORN domains [2]. Several other studies also show a relationship between the location of the mutation and onset of the disorder. Mutations occurring near the active sites cause neonatal onset, while those found on the protein surface usually cause late-onset OTCD [2, 3, 15, 16]. Experimental methods, such as enzymatic assays, enzyme kinetic studies, and functional assays, are used to detect the severity of the mutations; however, these methods are time consuming and costly [3].

In this study, we report two novel missense mutations in Malaysian patients with OTCD. To the best of our knowledge, we are the first to examine the potential impact of these mutations via in silico servers and molecular docking. These methods were also performed to known or reported* OTC* mutations, which were used for rational comparison.

## 2. Materials and Methods

### 2.1. Patients

Sixteen patients from several hospitals in Malaysia were referred to the Molecular Diagnostics & Protein Unit at the Institute for Medical Research for a molecular genetics study on OTCD. These patients presented with clinical signs of hyperammonemia and biochemical findings of OTCD (high blood ammonia, high plasma glutamine, low plasma citrulline, and high urinary excretion of orotate). To confirm that an alteration in the* OTC* gene was not a polymorphism, 50 blood samples from unrelated healthy individuals were also screened for the same mutation. Written informed consent was obtained from all patients and studied healthy individuals.

### 2.2. Molecular Analysis

#### 2.2.1. PCR Amplification

Genomic DNA was isolated from peripheral blood leukocytes using a QIAamp DNA blood kit (Qiagen, Valencia, USA). Polymerase chain reaction (PCR) was used to amplify all 10 exons of the* OTC* gene using 11 pairs of synthetic oligonucleotide primers, which were tagged with a universal M13 primer. After an initial denaturation of template DNA, amplification was performed using touchdown PCR [24]. The PCR product was purified using a QIAquick PCR purification kit (Qiagen, Valencia, USA), followed by cycle sequencing. Direct-cycle sequencing of all PCR fragments was performed according to the manufacturer's protocol. Bidirectional sequencing was conducted with Big Dye Terminator version 3.1 cycle-sequencing chemistries (Applied Biosystems, Fraser City, USA) on a 4-channel capillary ABI 3130-Avant Genetic Analyzer.

#### 2.2.2. Mutational Analysis

To identify the nucleotide and amino acid changes in the human* OTC* gene (accession no. NM_000531), DNA sequences were aligned and compared with cDNA sequences using SeqScape Software version 2.5 (Applied Biosystems, Foster City, USA). The detected mutations were identified as new or reported by comparing the results with mutations listed in the Human Genome Mutation Database (HGMD) (http://www.hgmd.cf.ac.uk/ac/index.php) [25] and 1000 Genome Database (http://browser.1000genomes.org) [26]. Numbering of DNA mutations was based on cDNA reference sequences, designating nucleotide + 1 as A of the ATG translation-initiation codon. Mutation nomenclature followed the recommendations of the Human Genome Variation Society (http://www.hgvs.org/mutnomen).

### 2.3. Bioinformatics Analysis

#### 2.3.1. Sequence Alignment Analysis

Multiple sequence alignment across species was performed using HomoloGene program from the National Centre for Biotechnology Information (NCBI) website (http://www.ncbi.nlm.nih.gov/homologene).

#### 2.3.2. Structure Selection and Identification of Active-Site Residues

Three-dimensional (3D) crystal structures of human OTCase were retrieved from the Protein Database Bank (PDB) (http://www.pdb.org) [27]. The crystal structures used in this study were crystallized with PALO ligand (PDB ID: 1OTH) [12], CP and NVA ligands (PDB ID: 1C9Y) [13], and CP ligand (PDB ID: 1EP9 and 1FVO) [14]. Identification of active-site residues was based on known residues that interact with the three ligands PALO, CP and NVA, and CP. The identified active-site residues were Ser90, Thr91, Arg92, Thr93, Arg141, Leu163, His168, Gln171, Asn199, Ile200, Asp263, Ser267, Met268, Cys303, Leu304, and Arg330.

#### 2.3.3. SNP Analysis and In Silico Prediction of Mutations

Known* OTC* missense mutations, located in the ligand-binding pocket, were compiled from the Human Genome Mutation Database (HGMD) [25] and 1000 Genome Browser [26]. The novel and known mutations were then assessed as disease-causing using PolyPhen 2.0 [28], SiFT [29], PhD-SNP [30], SNPs&GO [31], and MutPred [32].

PolyPhen 2.0 is based on the combination of sequence and structure-based attributes. It applies a naive Bayesian classifier for the identification of amino acid substitutions and impact of mutations. There are three possible output levels. These are probably damaging (0.85–1.0) and possibly damaging (0.15–0.84), which are classified as functionally significant, and the benign level (0–0.14) is classified as tolerated. SiFT is a sequence homology-based tool that sorts intolerant amino acid substitutions from the tolerant ones. In this program, a query sequence is analyzed by using multiple alignment information to predict tolerated and deleterious substitutions for every position of the query sequence. Positions with normalized probabilities ≤ 0.05 are predicted to be deleterious or damaging, and the score ≥ 0.05 is predicted to be tolerated. PhD-SNP is based on the support vector machine (SVM), which predicts the mutation based on protein sequence and profile information. The output classifies a mutation as a disease-related or neutral polymorphism using a reliability index. SNP&GO is also based on the support vector machine (SVM), which predicts deleterious SNPs using variations in protein sequence, and evolutionary information and function, as encoded in gene ontology terms. The output of the analysis predicts a disease-related polymorphism (Disease) if the score > 0.5 or as a neutral polymorphism (Neutral) if the score < 0.5. The result of the analysis provides information on variation probabilities for each protein associated with human disease. MutPred is based on SiFT with additional predictors of 14 different structural and functional properties. It uses a Random Forest classifier to provide a *g* score for SNPs predicted as disease associated (where the *g* is between 0.5 and 0.75); it also uses a* p* score to indicate which structural and functional properties may be impacted (where the *p* is between 0.01 and 0.05).

#### 2.3.4. Preparation of Protein and Ligand Structures for Molecular Docking

The native and mutant protein structures were prepared based on the OTCase monomer structure bound to PALO; the coordinates were acquired from the PDB (PDB ID: 1OTH). The OTCase monomer mutant models were generated via the Pymol program by retaining the water molecules and ligand from the crystal structure [33]. The tools in the Pymol program were used to substitute a single amino acid in the crystal structure of OTCase. For each mutant model, the top side-chain rotamer, assigned by Pymol, was chosen as the least pairwise overlap based on atomic van der Waals radii. The protein structures were submitted to the PDB2PQR server (version 2) (http://nbcr-222.ucsd.edu/pdb2pqr_2.0.0/) to add a missing hydrogen, resolve steric hindrance, optimize the hydrogen bond network, and assign residue protonation states at pH 7 [34]. The AMBER force field was used to assign the atomic charges to all the residues. The protonation state was based on the nitrogen epsilon of residue His168, in which this atom can form hydrogen bonds with the carbonyl oxygen of PALO in the crystal structure. This interaction majorly contributes to the catalytic mechanism.

The quality of the mutant models was assessed by ProSA-web. The ProSA-web calculates energy profiles (z-score) for modeled structures using molecular mechanics force field [35]. The z-score predicts overall model quality and measures the total energy deviation of the structure using random conformations. The modeled structure is predicted to be erroneous if the range of the z-scores falls outside that characteristic of native proteins. For the ligand, the 3D structure of PALO (PubChem ID: 124992) was retrieved from the PubChem database [36]. The ligand was converted from the SDF format to the PDB format using Open Babel [37]. The built mutant structures and the prepared ligand were used for the docking procedures.

#### 2.3.5. Docking

Initially, PALO was redocked to the native structure of human OTCase to verify the structure and obtain the standard-docking parameter. During the redocking process, optimization parameters (exhaustiveness and number of runs) were evaluated with respect to the native structure to obtain the highest binding affinity and correct conformation of PALO.

The AutoDock Tools 1.5.6 (ADT) program was used to prepare the input files for proteins (using the PQR format) and ligands (using the PDB format); both files were converted to PDBQT, the readable AutoDock Vina format [38, 39]. In addition, ADT was used to assign the grid box, which covered all the residues known to interact with the ligands; this was based on the crystal structure of PALO [12], CP-NVA [13], and CP [14] of human OTCase. The size of the grid box was 30 Å × 30 Å × 30 Å, with the spacing of 1.0 Å centered at the coordinates x, y, and z (0.443, 0.612, and −25.00). Subsequently, both protein and ligand files were submitted to AutoDock Vina for docking calculation; exhaustiveness was set at 8 as the optimized parameter, and the number of runs for the search ligand conformation was set at 9. The protein was kept rigid and the ligand was allowed to vary. The docking result was evaluated using the binding affinity score (kcal/mol). The best ligand conformation was ranked based on the lowest docked energy. The conformation of the PALO ligand was then superimposed onto the PALO ligand in the crystal structure. Pymol was used to visualize the ligand-binding modes, analyze the root mean standard deviation (RMSD), and observe hydrogen bond interactions between the protein and the ligand [33, 40]. The same procedures were performed to analyze the mutant structures.

## 3. Results

### 3.1. Clinical and Molecular Analyses

Table 1 shows the clinical findings on 16 patients with OTCD from 14 unrelated families. The patient cohort consisted of 11 males with neonatal onset OTCD and five symptomatic females. All the 11 males with neonatal onset OTCD presented acute encephalopathy at the age of 2.7 ± 0.12 days (range, 2–5 days). Their maximum blood ammonia at presentation was 1,298 ± 719 *μ*mol/L. Eight of these male patients died during their first hyperammonemic encephalopathy despite nutritional support, administration of intravenous ammonia scavengers, and intensive care. Three male patients survived the neonatal period. Two of them had recurrent metabolic decompensations and died at the ages of 6 years (Patient 1) and 2 years (Patient 12). Another patient (Patient 13) had mild psychomotor retardation when last reviewed at 3 years and 6 months of age. The five symptomatic females with heterozygous mutations in the OTC gene presented with vomiting and altered sensorium at the age of 11.0 ± 14.0 years (range, 0.02–34 years). Their maximum blood ammonia at presentation was 400 ± 144 *μ*mol/L. Two of them died during the initial hyperammonemic episode.

**Figure 1:**
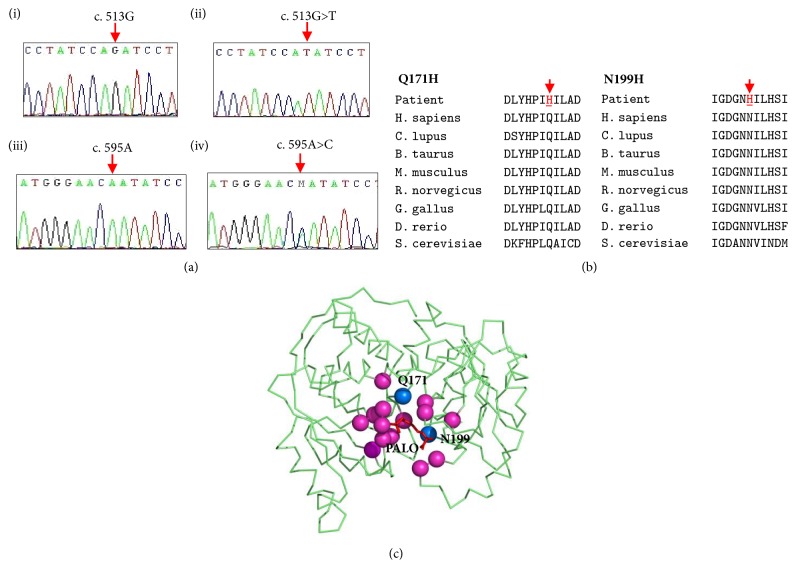
(a) DNA sequencing profiles based on electropherograms; ((i) and (iii)) native human OTCase, (ii) homozygous mutation at c.513G>T, and (iv) heterozygous mutation at c.595A>C. (b) Multiple alignment of human OTC with seven other species. The data reveals that histidine and asparagine at positions 171 and 199, respectively, are highly conserved among other species. Mutant residues are indicated as red arrow. (c) Distribution of 16 residues represents 32 SNPs occurring at the ligand-binding pocket in human OTCase studied in this work. Novel mutations found in this study; Q171 and N199 (blue) and other mutations (magenta).

Molecular analysis identified 13 different mutations.  Table 2 shows 11 missense mutations and 2 small deletions detected among the 16 patients. In these 13 mutations, we identified 2 novel mutations, Q171H and N199H, while the other 11 mutations were already reported. The results were validated based on the absence of mutations in 50 unrelated healthy individuals from the studied population. The first novel mutation, Q171H, which was found in Patient 1, occurred due to the substitution of G into T in codon 513 of exon 5 (Figure 1(a)-(ii)). The second novel mutation, N199H, found in Patient 2, occurred due to the substitution of A into C in codon 595 of exon 6 (Figure 1(a)-(iv)). The novel mutations were then further evaluated using multiple sequence alignments. In both cases, the alignment results showed that Q171 and N199 were conserved in seven other species (Figure 1(b)). Based on the crystal structures of human OTCase, we found that these residues were located in the core of the ligand-binding pocket (Figure 1(c)). Therefore, we also compiled the known missense mutations located in the ligand-binding pocket of OTCase, using HGMD and 1000 Genome databases for rational comparison. Our key aim was to identify whether these mutations exert similar or different effects on the structure of OTCase. To date, we found 30 mutations, which were located at 16 residues (Figure 1(c)).

### 3.2. In Silico Assessment of Novel and Known OTCase Missense Mutations

We further studied the effects of the novel mutations, Q171H and N199H, as well as those of 30 known mutations, using five in silico prediction servers. The five in silico servers showed consensus in predicting 17 mutations, while four in silico servers showed consensus in predicting 15 mutations (Supplementary Table S1). The PolyPhen server predicted damaging effects for 31 mutations, while the SiFT server predicted damaging functional effects for all the mutations. The PhD-SNP and SNP&GO servers indicated 32 and 29 mutations, respectively; these mutations were predicted to be disease-related. Subsequently, the MutPred server indicated 21 mutations, which were deleterious/disease-related.

All five in silico servers predicted that both novel mutations would cause structural and functional defects in the OTCase. Both PolyPhen and SiFT predicted the Q171H mutation as damaging, while PhD-SNP and SNP&GO predicted it as disease-related. The MutPred server generated a (*g*) score of 0.852 with a* p* score of 0.0246; this confidence hypothesis indicates that the mutation will affect protein function. Another known mutation, Q171E, located at the same residue position, generated similar results. For the novel mutation N199H, PolyPhen and SiFT predicted this mutation as damaging, while PhD-SNP and SNP&GO predicted it as disease-related. Although the results of MutPred generated a (*g*) score of 0.945, indicating a highly deleterious effect, the* p* score was absent, and confidence hypothesis was not obtained from prediction reliability. Similar results were obtained for the other two known mutations at the same residue position; these were N199D (*g* = 0.989) and N199S (*g* = 0.988), with low confidence hypothesis.

### 3.3. Mutant Models and Docking of PALO

**Figure 2:**
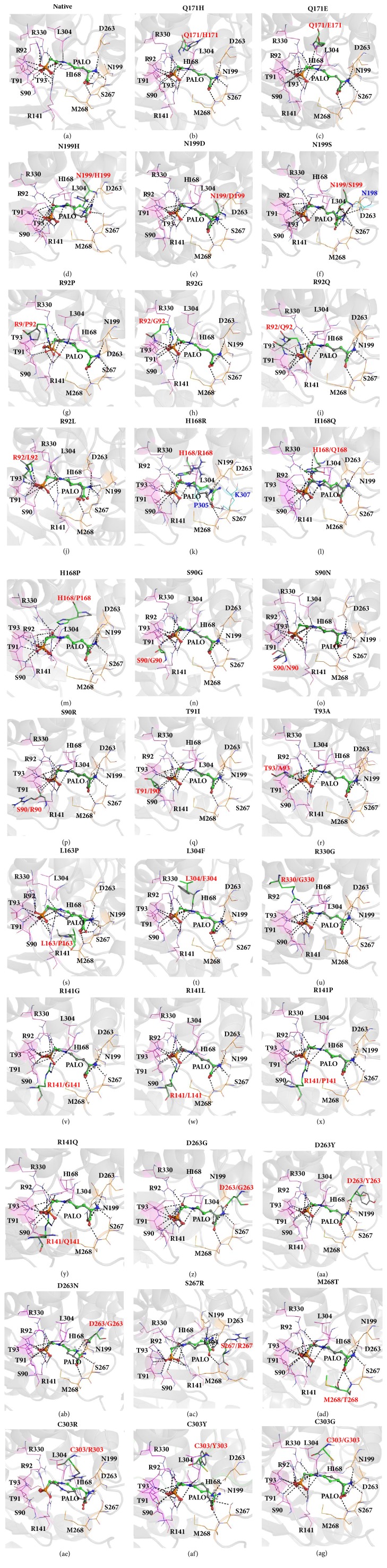
Superimposition between mutant and native structures and hydrogen bond interactions between PALO and residues in CP and ORN binding domains in the native and mutant structures. PALO in the native and mutant structures is shown as green and grey balls and sticks, respectively. Residues in the native and mutant structures are shown as green and grey stick, respectively. New residues interacting with PALO are shown in cyan line. Residues in CP binding domain are shown in magenta line, while residues in ORN binding domain are shown in orange line.

The qualities of the mutant models were performed using ProSA-web. The z-scores of all the mutant models were negative and within the range of the score of the native protein, confirming the absence of problematic parts (Supplementary Table S2). Molecular docking was used to observe how the two novel and 30 known mutants affect ligand binding in the structures of OTCase. Initially, we conducted redocking of the crystal structure of the OTCase monomer (PDB ID: 1OTH) [12]. Superimposition of the docked PALO from the native structure and the PALO from the crystal structure produced an RMSD of 0.11 Å and binding affinity score of −8.4 kcal/mol. In this native structure, the docked PALO interacted with 12 residues in the ligand-binding pocket. The CP-PALO directly interacted with eight residues in the CP binding domain; these were S90, T91, R92, T93, R141, H168, L304, and R330. The ORN-PALO directly interacted with four residues in the ORN binding domain; these were N199, D263, S267, and M268 (Figure 2(a)).

The results of PALO docking to 32 mutant models indicated that PALO bound to the ligand-binding pocket with binding affinities between −6.5 and −8.4 kcal/mol (Supplementary Table S2). The novel mutation, Q171H, presented the same PALO binding affinity as that of the native structure (−8.4 kcal/mol). Superimposition of docked PALO onto the native structure produced an RMSD of 0.11 Å. The substituted H171 showed that all the residues in the binding pockets formed the same interactions as those in the native structure (Figure 2(b)). However, the substituted H171 lost interaction with the neighboring residues, E326 and R330. For comparison, a known mutation, Q171E, found at the same position, generated the same PALO binding affinity as those generated by novel and native structures. Superimposition of docked PALO onto the native structure produced an RMSD of 0.12 Å. This known mutation did not affect the conformation of PALO, in which all the residues in the binding pocket interact with PALO (Figure 2(c)). Nevertheless, the replacement of glutamine with glutamate produced a loss of interactions with only one neighboring residue, E326.

The binding affinity for PALO of another novel mutation, N199H, was slightly lower than that of the native structure (−7.8 kcal/mol). Superimposition of docked PALO onto the native structure produced an RMSD of 1.74 Å. The aromatic ring of the substituted histidine caused small conformational changes to the ORN-PALO. The substituted H199 showed no interaction with the ORN-PALO (Figure 2(d)). Concurrently, this substituted residue also lost interactions with two neighboring residues, S164 and I200. However, this substituted residue maintained a hydrogen bond with another neighboring residue, S203. In addition, there were two known mutations at the position N199: N199D and N199S. These mutant models generated PALO binding affinities of −8.1 and −8.3, respectively; these affinities were similar to that of the native structure. Superimposing the docked PALO from the N199D and N199S mutants to the native structure produced RMSDs of 0.21 Å and 0.55 Å, respectively. The substituted S199 showed that ORN-PALO formed an additional interaction with N198 (Figure 2(e)), while the substituted D199 retained the same interactions as the native structure (Figure 2(f)). These two known mutations also caused loss of interactions with two neighboring residues, S164 and I200. However, the interaction with another neighboring residue, S203, was maintained; this was similar to the interactions of the native and novel mutation, N199H.

Among the mutants located at the CP binding domain, R92P and H168R exhibited the lowest binding affinities. Docking of PALO to the mutant R92P exhibited the lowest binding affinity (−6.5 kcal/mol). Superimposition of docked PALO from R92P mutant onto the native structure produced an RMSD of 0.342 Å. A substitution of arginine with proline caused conformational changes in CP-PALO; the phosphate oxygen atoms (O2P and O1P) of CP-PALO lost interactions with P92 and T93 (Figure 2(g)). However, other mutants at the positions R92, R92G, R92Q, and R92L exhibited binding affinities that fell between −7.9 and −8.1 kcal/mol. Superimposition of the docked PALO from the R92G, R92Q, and R92L mutants onto the native structure produced RMSDs of 0.119 Å, 0.152 Å, and 0.237 Å, respectively. The PALO in R92G and R92Q mutants behaved structurally similar to the native structure (Figure 2(h) and Figure 2(i)). However, the substitution of arginine with leucine caused the carbonyl oxygen atom of this PALO to lose interaction with the hydrogen delta of N199 (Figure 2(j)). However, these mutations did not affect the hydrogen bond interactions with neighboring residues.

The position at residue H168 had three known mutations, H168R, H168Q, and H168P. The docking of PALO to the H168R mutant produced the second lowest binding affinity score (−6.9 kcal/mol); the RMSD of PALO to the native structure was 2.02 Å. The results of docking analysis showed that PALO did not interact with the residues R168, T93, N199, and S267 (Figure 2(k)). In addition, PALO formed new interactions with P305 and K307 (Figure 2(k)). The other two mutants at the positions H168, H168Q, and H168P produced binding affinities of −7.6 and −8.1 kcal/mol, respectively. Superimposition of the docked PALO from the mutants H168Q and H168P onto the native structure produced RMSDs of 0.171 Å and 0.511 Å, respectively. The PALO docked to the mutant H168Q behaved structurally similar to the native structure (Figure 2(l)). However, the substitution of proline with histidine caused PALO to lose interaction with P168 (Figure 2(m)). Concurrently, the substituted R168, Q168, and P168 did not affect interactions with neighboring residues.

Three mutations near the residue R92, which were S90, T91I, and T93A, exhibited PALO binding affinities of −8.3, −8.4, and −7.5 kcal/mol, respectively. Superimposition of the docked PALO from the mutants S90G, S90N, and S90R onto the native structure produced RMSDs of 0.148 Å, 0.143 Å, and 0.171 Å, respectively. The results of docking analysis showed that PALO did not interact with the substituted residues G90, N90, and R90 (Figures 2(n), 2(o), and 2(p)). In addition, the substituted residues G90, N90, and R90 lost interactions with the neighboring residue R94. The mutants T91I and T93A did not alter the ability of the residues in the ligand-binding pocket to form interactions with PALO (Figures 2(q) and 2(r)). However, the substituted A93 lost its interactions with the neighboring residues R141 and R330.

The binding affinities of other three known mutations, L163P, L304F, and R330G, were >−8.0 kcal/mol. Substitution of leucine with proline caused PALO to lose interaction with L304 (Figure 2(s)). This replacement also caused the substituted P163 to lose its interaction with the neighboring residue N161. Nevertheless, the PALO in the L304F mutant behaved structurally similar to the native structure (Figure 2(t)). Superimposition of the docked PALO from mutant L304F onto the native structure produced an RMSD of 0.130 Å. Substitution of leucine with phenylalanine did not affect interactions with neighboring residues. Unlike the replacement of arginine with glycine (R330G), PALO lost interactions with substituted G330 (Figure 2(u)). The substituted G330 also lost interactions with the two neighboring residues T93 and Q171. The other four known mutations at the residue R141, which are R141G, R141L, R141P, and R141Q, exhibited PALO binding affinities between −7.6 and −7.7 kcal/mol. Superimposition of the docked PALO from the mutants R141G, R141L, R141P, and R141Q onto the native structure produced RMSDs of 0.110 Å, 0.176 Å, 0.140 Å, and 0.117 Å, respectively. The docking results showed that PALO did not interact with the substituted residues G141, L141, P141, and Q141 (Figures 2(v), 2(w), 2(x), and 2(y)). In addition, these substituted residues also lost interactions with the three neighboring residues R89, T93, and G162.

Three residues in the ORN binding domain, D263, S267, and M268, contained known mutations. Three mutations, D263G, D263Y, and D263N, found at residue 263, exhibited binding affinities between −7.1 and −8.2 kcal/mol. Superimposition of the docked PALO from mutants D263G, D263Y, and D263N onto the native structure produced RMSDs of 0.131 Å, 0.141 Å, and 1.738 Å, respectively. The docking results showed that PALO lost interactions with the two substituted residues G263 and Y263 (Figures 2(z) and 2(aa)). Substitution of aspartate with asparagine (D263N) caused PALO to lose its interactions with the residue N199 (Figure 2(ab)). The substituted N263 caused PALO to form a new interaction with S267. Mutations at S267R and M268T exhibited binding affinities of −8.0 kcal/mol. Superimposition of the docked PALO from the mutants S267R and M268T onto the native structure produced RMSDs of 0.731 Å and 0.168 Å, respectively. Substitution of serine with arginine at residue 267 caused PALO to lose its interactions with the substituted R267 (Figure 2(ac)). However, we observed that the PALO docked to the substituted T268 behaved structurally similar to the native structure (Figure 2(ad)). The substituted R267 and T268 did not affect interactions with the neighboring residues.

Another mutation occurred at the residue C303. This residue did not directly interact with PALO; however, this residue is involved in the OTCase catalytic activity. This residue had three known mutations: C303R, C303Y, and C303G. The results of docking to the mutants showed binding affinities of −8.0 to −8.3 kcal/mol; these affinities were similar to that of the native structure. The substituted R303 caused PALO to lose interactions with the residues H168 and R330; however, PALO concurrently formed a new interaction with residue D263 (Figure 2(ae)). The substituted R303 formed an interaction with the neighboring residue H168. In the case of substituted Y303, PALO formed a new interaction with S267 (Figure 2(af)). This substituted residue also formed an interaction with the neighboring residue D175. In the case of substituted G303, the docked PALO behaved structurally similar to the native structure (Figure 2(ag)).

## 4. Discussion

In this study, we reported two novel mutations, Q171H and N199H, found in Malaysian patients with OTCD. These two mutations were located at 16 residues in the OTCase ligand-binding pocket; these residues formed direct or indirect interactions with the cocrystallized ligands (PALO, CP and NVA, and CP) of the three human OTCase crystal structures [12–14]. Compiling the known* OTC* mutations from the HGMD and 1000 Genome databases resulted in 30 reported missense mutations. These 30 known missense mutations were also found at these 16 residues in the catalytic pocket. In contrast, a study by Magesh et al. (2014) only found 19 known missense mutations located at the ligand-binding pocket [41]. The high number of missense mutations, identified in this study, indicates that advances in sequencing technology have increased the likelihood of identifying mutations.

Although a different in silico server, MutPred, was used in our study as opposed to the I-Mutant 3.0 used by Magesh et al. [41], our findings were consistent with their findings. In our study, the five in silico servers successfully predicted the two novel and 30 known mutations as disease-causing. The comparison of the novel mutant Q171H with the known mutant Q171E indicates that the in silico server successfully generated the same consensus. Similar to the prediction of the novel mutant N199H, the in silico server successfully generated the same consensus on the two known mutants, N199D and N199S. This study shows that using more than one server for predicting mutations can increase prediction consensus and improve the accuracy of distinguishing damaging mutations from neutral ones. These results also support a previous prediction, stating that missense mutations located at the catalytic pocket are the main cause of severe OTCD [3].

The docking analysis was first performed with the native and mutant OTCase monomer structures. The results showed that the redocking of the native monomer structure was similar to that of the OTCase monomer crystal structure, in which all active-site residues in CP and ORN binding domains formed interactions with CP-PALO and ORN-PALO [12]. The docking analysis was then performed with both novel* OTC* mutations using rational comparison with 30 other known* OTC *mutations. The results indicated that PALO bound to the ligand-binding pocket of all the mutants but showed small conformational changes compared to the binding to the native structure. In the case of the Q171H mutation, the docking of PALO to the mutant model showed no conformational or binding affinity changes of the ligand compared with those of the native structure. As in the crystal structure, Q171H did not interact with PALO. However, the substitution of glutamine with histidine disturbed interactions among several residues in the binding pocket; these residues included R330, which forms interactions with the carbonyl oxygen of CP-PALO [12]. Because CP was the first substrate that entered into the catalytic site, we speculated that the disturbed interaction between residue R330 and CP-PALO may largely affect the stability of the pocket, subsequently impacting catalytic activity. The effects were evident in one of the male patients in this study (Patient 1), who survived the neonatal stage but died at the age of 6 years with recurrent metabolic decompensations.

In the case of mutation N199H, the docking of PALO to the mutant model slightly affected the binding affinity and conformation of PALO. In the native structure, the N199 residue directly interacted with PALO, forming hydrogen bonds with both carboxyl and alpha-amino groups of the ORN-PALO domain. In the mutant model, the substituted H199 lost interactions with ORN-PALO and other neighboring residues. Because of N199H, located in the ORN binding domain, this disruption may affect the binding of ORN-PALO, the second substrate for catalytic activity. In addition, the stability of the structure may be affected because of the change in the bonding pattern. The female patient with the N199H mutation (Patient 2), diagnosed with late-onset OTCD, demonstrated mild symptoms. However, her male sibling lapsed into hyperammonemic coma and died on day 40, based on family history. This indicates that mutations at this position can potentially cause severe effects, especially in male patients, because this X-linked disease affects males more than females. Mutations at the same residue positions N199S are also found in neonatal onset OTCD [2, 3, 23] and at N199D in female patients [2, 3].

We will now discuss other mutations with respect to the CP and ORN binding domains. The CP binding domain is located in the core of the ligand-binding pocket, where the residues are the most conserved and the least exposed to solvents [3]. A study by Caldovic [3] showed that most missense mutations occur in this domain [3]. Among the 30 known mutations, we found that R92P, followed by H168R, had the most impact on ligand conformation and produced the lowest binding affinities to PALO. Most of the mutations at R92 are reported in neonates, while mutations at H168 are found in females or late-onset cases [2, 3]. R92 and H168 belong to STRT and HPxQ motifs, respectively [12, 13, 42]. In addition, both residues are highly conserved in all OTCase structures [12, 42]. Therefore, mutations at these positions are highly likely to affect ligand binding; they may also affect the stability of the structure, because both residues are important for CP binding [14, 42].

The other mutations at the CP binding domain were located at S90, T91, T93, R141, L304, and R330. Although mutations at these positions did not affect the conformation of PALO, they produced slightly lower binding affinities compared to that of the native structure. These mutations were also found to alter the bonding pattern with CP-PALO and surrounding residues. Furthermore, mutations at positions S90, T91, and T93 belong to the STRT motif, while L304 belongs to the HCLP motif [12, 42]. The residues at these positions are conserved in all OTCase structures [12, 42]. Hence, mutations occurring at these positions may impact the stability of the structure by affecting hydrogen bond interactions with CP-PALO or the neighboring residues. OTCD resulting from mutations at these residue positions is reported in neonates, late-onset OTCD, and females [2, 3].

In the ORN binding domain, there were three residues: D263, S267, and M268. For residue D263, the D263N mutant showed the lowest binding affinity score. However, all the mutants at this residue lost hydrogen bonds with ORN-PALO or several neighboring residues. The other two mutants, S267R and M268T, exhibited same behavior as that of the native structure. Nevertheless, the substituted R267 did not form any interactions with ORN-PALO. These three residue positions are part of the DxxxSMG motif, which may interact with the second substrate in the structure of OTCase [12, 42]. Residues at these positions are conserved among OTCase structures [12, 13, 42]. Thus, mutations occurring at these positions likely disrupt the stability of the OTCase structure by affecting interaction with ORN-PALO. In addition, OTCD related to these mutations is found in late-onset OTCD and females [3].

The results of docking analysis indicated that the binding affinity of the mutant model C303 was similar to that of the native structure. However, the substituted residues R303 and Y303 affected the bonding pattern between PALO and the neighboring residues. Although C303 does not directly interact with PALO in the crystal structure, this residue is part of the HCLP motif and plays a role in the catalytic reaction. Therefore, mutations at this position are highly likely to affect the function of OTCase. Furthermore, the two reported cases of OTCD with mutations at residue C303 presented with neonatal onsets, and only one of these occurred in a female [3, 23, 43, 44].

## 5. Conclusion

In this study, in silico servers were used to predict that all novel and known mutations were pathogenic. Docking analysis showed that PALO could bind to the catalytic site of the OTCase mutants. Missense mutations caused small conformational changes in PALO and disrupted interactions between the residues in the binding pocket and the ligand; these mutations also disrupted interactions among the residues. Hence, these mutations impact the structure, which can subsequently affect the catalytic activity.

## Figures and Tables

**Table 1:** Summary of clinical and molecular findings of 16 patients with OTC deficiency.

**Patient**	**Sex**	**First acute episode**					**Subsequent clinical progress **	**Family history**
		**Age **	**Presenting symptoms**	**Blood ammonia (first measurement - maximum value, *μ*mol/l, N.R. 50–80) **	**Therapy received **	**Outcome**		
1	M	3d	Poor suck, reduced oral intake and lethargy progressing to coma	350 -780	Stop protein intake, PFN, SB, SPB, L-Arg, PD, MV	Survived	Spastic tetraplegia, severe psychomotor retardation, frequent hyperammonemic episodes despite a low-protein diet and treatment with oral ammonia scavengers, fatal encephalopathy at 6 y	Negative

2	F	6y	Recurrent vomiting, difficulty in breathing following a febrile illness	215 - 310	iv SB, SPB, L-Arg,	Survived	Mild learning disability, infrequent decompensation on a low-protein diet and oral ammonia scavengers	A younger male sibling died of hyperammonemic coma at 40d

3	M	2d	Poor feeding, lethargy, tachypnea, irritability, coma	305 - 850	Stop protein intake, PFN, PD, MV	Died	-	Negative

4	F	7d	Feeding intolerance, irritability, altered sensorium	250 - 480	Stop protein intake, PFN, SB, SPB, L-Arg, PD, MV	Survived	Moderate developmental delay/learning disability, recurrent decompensation	Negative

5	M	5d	Sudden onset of fits, progressing to somnolence and coma and respiratory arrest	450 – 2,361	Stop protein intake, PFN, MV	Died	-	A male sibling died suddenly during neonatal period (undiagnosed)

6	M	2d	Feeding refusal, vomiting, lethargy, progressing to coma	750 - 865	Stop protein intake, PFN, MV	Died	-	Negative

7	M	3d	Poor suck, lethargy, hypotonia, coma	345 – 2,915	Stop protein intake, PFN, PD, MV	Died	-	Negative

8	F	1y	History of failure to thrive, hypotonia, seizures. Acute encephalopathy and respiratory distress following a febrile illness.	236 - 560	Stop protein intake, PFN, phenytoin, MV	Died	-	Multiple neonatal deaths among maternal male siblings

9	M	5d	Vomiting, lethargy, seizures, progressing to coma	201 - 990	Stop protein intake, PFN, SB, SPB, L-Arg, PD, MV	Died	-	Negative

10	M	2d	Poor suck, vomiting, progressive lethargy, and irritability	320 - 980	Stop protein intake, PFN, MV	Died	-	Negative

11	M	2d	Lethargy, poor breathing effort, progressing to cardiorespiratory collapse	1,700	Stop protein intake, PFN, MV	Died	-	Multiple neonatal deaths among maternal male siblings

12	M	2d	Poor oral intake and lethargy progressing to coma	468 - 789	Stop protein intake, PFN, SB, SPB, L-Arg, PD, MV	Survived	Severe psychomotor retardation, frequent hyperammonemic episodes despite a low-protein diet and treatment with oral ammonia scavengers, fatal encephalopathy at 2y	Negative

13	M	2d	Reduced oral intake, lethargy, irritability, progressing to coma	248 – 1,065	Stop protein intake, PFN, SB, SPB, L-Arg, CVVH, MV	Survived	Mild psychomotor retardation, infrequent hyperammonemic episodes on low-protein diet and oral ammonia scavengers	Negative

14	M	2d	Poor suck, lethargy, abnormal breathing, progressing to coma	332 - 976	Stop protein intake, PFN, SB, SPB, L-Arg, MV	Died	-	First one diagnosed in the family

15	F	14y	Vomiting, seizures and progressive acute encephalopathy following a febrile illness	Not done	PFN, phenytoin, antibiotics, MV	Died	-	Sibling of Patient 14 (They presented around the same time.)

16	F	34y	History of protein avoidance. Post-partum delirium	180 - 250	Stop protein intake, PFN, high calories intake, SB, SPB, L-Arg	Survived	Mildly symptomatic. Nausea if consuming high protein content foods	Mother of Patients 14 and 15

*M: *male, *F: *female, *y:* years, *d: *days, *PFN:* parenteral fluid and nutrition, *L-Arg:* L-arginine, *SB:* sodium benzoate, *SPB:* sodium phenylbutyrate, *PD:* peritoneal dialysis, *CVVH: *continuous venovenous hemodiafiltration, and *MV:* mechanical ventilation.

**Table 2:** List of mutations found in 16 patients.

Patient (Sex)	Exon/ Intron	Nucleotide change	Amino acid change	Reference
1 (male)	Ex-5	c.513G>T	p.(Gln171His)	**Novel** ^**a**^
2 (female)	Ex-6	c.595A>C	p.(Asn199His)	**Novel** ^**a**^
3 (male)	Ex-2	c.133C>G	p.(Leu45Val)	[17]
4 (female)	Ex-5	c.422G>A	p.(Arg141Gln)	[18]
5 (male)	Ex-4	c.299 G>A	p.Gly100Asp)	[19]
6 (male)	Ex-3	c.286T>C	p.(Ser96Pro)	dbSNP database
7 (male)	Ex-5	c.422G>A	p.(Arg141Gln)	[18]
8 (female)	Ex-8	c.813-814delAGinsC	p.(Glu271Aspfs*∗*288)	[20]
9 (male)	Ex-6	c.595A>G	p.(Asn199Asp)	[2]
10 (male)	Ex-6	c.583G>A	p.(Gly195Arg)	[21]
11 (male)	Ex-2	c.148 T>G	p.(Gly50*∗*)	[22]
12 (male)	Ex-10	c.245_246delTAinsAG	p.(Leu82*∗*)	[4]
13 (male)	Ex-9	c.1005G>A	p.(Met335Ile)	[23]
14 (male)	Ex-10	c.638T>C	p.(Met213Thr)	[3]
15 (female)	Ex-10	c.638T>C	p.(Met213Thr)	[3]
16 (female)	Ex-10	c.638T>C	p.(Met213Thr)	[3]

## Data Availability

The data used to support the findings of this study are included within the article.

## Abbreviations

OTCD: Ornithine transcarbamylase deficiency
PALO: N-(Phosphonoacetyl)-L-ornithine
ORN: Ornithine
CP: Carbamoyl phosphate
OTC: Ornithine transcarbamylase
SNPs: Single nucleotide polymorphisms
PCR: Polymerase chain reaction
DNA: Deoxyribonucleic acid
cDNA: Complementary DNA
HGMD: Human genome mutation database
OMIM: Online Mendelian Inheritance in Man
NCBI: National Centre for Biotechnology Information
PolyPhen 2: Polymorphism Phenotyping version 2
SiFT: Sorting Intolerant From Tolerant
PhD-SNP: Predictor of human Deleterious Single Nucleotide Polymorphisms
SNP&GO: Predicting disease associated variations using GO terms
SVM: Support vector machine
PDB: Protein Database Bank
RMSD: Root mean score deviation.

## Supplementary Materials

Supplementary Materials Table S1: in silico prediction of novel and known mutations located at ligand-binding pocket in the OTCase structure. Table S2: Z-score, binding affinities, RMSDs, and hydrogen bond interactions of native and mutant models.
