# Supplementary material for: Mutation Study of Malaysian Patients with Ornithine Transcarbamylase Deficiency: Clinical, Molecular, and Bioinformatics Analyses of Two Novel Missense Mutations of the OTC Gene
Source: Biomed Res Int. 2018 Aug 5;2018:4320831. doi: 10.1155/2018/4320831 (PMC6098936; doi:10.1155/2018/4320831)
Supplement: Supplementary Materials — Table S1: in silico prediction of novel and known mutations located at ligand-binding pocket in the OTCase structure. Table S2: Z-score, binding affinities, RMSDs, and hydrogen bond interactions of native and mutant models. [file 4320831.f1.docx]

**Supplementary data for the manuscript:**

**Mutation Study of Malaysian Patients with Ornithine Transcarbamylase Deficiency: Clinical, Molecular and Bioinformatics Analyses of Two Novel Missense Mutations of the *OTC* Gene using *In Silico* Servers and Molecular Docking Method**

**Ernie Zuraida Ali^1,2,^*, Yuslina Zakaria^1^, Mohd Amran Mohd Radzi^3^*,* Ngu Lock Hock^4^ and Siti Azma Jusoh^1,^***

^1^Molecular Diagnostics and Protein Unit, Specialized Diagnostics Centre, Institute for Medical Research (IMR), Jalan Pahang, 50588 Kuala Lumpur, Malaysia.

^2^Faculty of Pharmacy, Universiti Teknologi MARA (UiTM), Puncak Alam Campus, 42300 Bandar Puncak Alam, Selangor, Malaysia.

^3^Department of Electrical and Electronic Engineering, Universiti Putra Malaysia, 43400 UPM Serdang, Selangor, Malaysia.

^4^Genetics Department, Kuala Lumpur Hospital, Jalan Pahang, 50586 Kuala Lumpur, Malaysia.

Correspondence should be addressed to Ernie Zuraida Ali; [erniez1980@gmail.com.my](mailto:erniez1980@gmail.com.my) and Siti Azma Jusoh; [sitiazma@puncakalam.uitm.edu.my](mailto:sitiazma@puncakalam.uitm.edu.my)

TABLE S1***:*** *In silico* prediction of novel and known mutations located at ligand binding pocket in the OTCase structure.

| **No** | **Dataset SNP from** | **Mutation** | **PolyPhen 2.0** | | **SiFT** | | **PhD-SNP** | **SNP&GO** | **MutPred** | | | | **No of server** |
| --- | --- | --- | --- | --- | --- | --- | --- | --- | --- | --- | --- | --- | --- |
|  |  |  | **PSIC** | **Prediction** | **Score** | **Prediction** |  |  | **G score** | **P score** | **Molecular Variation** | **Prediction reliability** |  |
| 1 | 1000 & HGMD | R92G | 0.848 | Possibly Damaging | 0 | Damaging | Disease | Disease | 0.992 | 0.0393 | Gain of ubiquitination at K88, Loss of catalytic residue at R92 | Confident Hypotheses | 5 |
| 2 | 1000 & HGMD | R92Q | 0.996 | Probably Damaging | 0 | Damaging | Disease | Disease | 0.994 | 0.0337 | Gain of ubiquitination at K88 | Confident Hypotheses | 5 |
| 3 | 1000 & HGMD | R92L | 0.998 | Probably Damaging | 0 | Damaging | Disease | Disease | 0.991 | 0.0289 | Gain of ubiquitination at K88, Loss of disorder, Loss of catalytic residue at R92 | Confident Hypotheses | 5 |
| 4 | 1000 & HGMD | R92P | 0.999 | Probably Damaging | 0 | Damaging | Disease | Disease | 0.982 | 0.0077 | Loss of catalytic residue at R92 | Very Confident Hypotheses | 5 |
| 5 | 1000 & HGMD | R141G | 1 | Probably Damaging | 0 | Damaging | Disease | Disease | 0.985 | 0.0272 | Gain of catalytic residue at V142, Loss of stability, Gain of sheet | Confident Hypotheses | 5 |
| 6 | 1000 | R141L | 1 | Probably Damaging | 0 | Damaging | Disease | Disease | 0.947 | 0.0344 | Gain of sheet | Confident Hypotheses | 5 |
| 7 | 1000 | L163P | 1 | Probably Damaging | 0 | Damaging | Disease | Disease | 0.944 | 0.0327 | Loss of stability, Gain of disorder | Confident Hypotheses | 5 |
| 8 | 1000 & HGMD | H168P | 1 | Probably Damaging | 0 | Damaging | Disease | Disease | 0.983 | 0.0116 | Gain of catalytic residue at H168 | Confident Hypotheses | 5 |
| 9 | HGMD | Q171E | 0.999 | Probably Damaging | 0 | Damaging | Disease | Disease | 0.838 | 0.0166 | Loss of catalytic residue at Q171 | Confident Hypotheses | 5 |
| 10 | Novel Mutation | Q171H | 1 | Probably Damaging | 0 | Damaging | Disease | Disease | 0.852 | 0.0246 | Loss of catalytic residue at Q171 | Confident Hypotheses | 5 |
| 11 | 1000 | D263Y | 1 | Probably Damaging | 0 | Damaging | Disease | Disease | 0.907 | 0.0221 | Gain of sheet | Confident Hypotheses | 5 |
| 12 | 1000 & HGMD | D263G | 1 | Probably Damaging | 0 | Damaging | Disease | Disease | 0.986 | 0.0086 | Loss of catalytic residue at D263 | Very Confident Hypotheses | 5 |
| 13 | 1000 & HGMD | S267R | 0.999 | Probably Damaging | 0 | Damaging | Disease | Disease | 0.968 | 0.0312 | Gain of MoRF binding | Confident Hypotheses | 5 |
| 14 | 1000 & HGMD | M268T | 0.994 | Probably Damaging | 0 | Damaging | Disease | Disease | 0.942 | 0.0055 | Loss of catalytic residue at M268 | Very Confident Hypotheses | 5 |
| 15 | 1000 & HGMD | C303R | 0.994 | Probably Damaging | 0 | Damaging | Disease | Disease | 0.984 | 0.0065 | Gain of MoRF binding | Very Confident Hypotheses | 5 |
| 16 | 1000 & HGMD | C303G | 0.996 | Probably Damaging | 0 | Damaging | Disease | Disease | 0.994 | 0.0249 | Gain of disorder | Confident Hypotheses | 5 |
| 17 | 1000 & HGMD | L304F | 0.96 | Probably Damaging | 0 | Damaging | Disease | Disease | 0.971 | 0.0151 | Loss of catalytic residue at L304 | Confident Hypotheses | 5 |
| 18 | 1000 & HGMD | S90G | 0.999 | Probably Damaging | 0 | Damaging | Disease | Neutral | 0.98 | 0.0051 | Loss of catalytic residue at S90 | Very Confident Hypotheses | 4 |
| 19 | 1000 & HGMD | S90N | 0.998 | Probably Damaging | 0 | Damaging | Disease | Neutral | 0.987 | 0.0326 | Loss of phosphorylation at S90, Loss of catalytic residue at S90 | Confident Hypotheses | 4 |
| 20 | 1000 & HGMD | S90R | 1 | Probably Damaging | 0 | Damaging | Disease | Neutral | 0.976 | 0.0251 | Loss of catalytic residue at S90, Loss of phosphorylation at S90 | Confident Hypotheses | 4 |
| 21 | 1000 | T91I | 0.321 | Benign | 0.01 | Damaging | Disease | Disease | 0.811 | 0.0189 | Loss of phosphorylation at T91, Loss of disorder | Confident Hypotheses | 4 |
| 22 | 1000 & HGMD | T93A | 0.912 | Probably Damaging | 0 | Damaging | Disease | Disease | 0.97 | 0.062 | Loss of phosphorylation at T93 | No reliable reference | 4 |
| 23 | 1000 & HGMD | R141Q | 1 | Probably Damaging | 0 | Damaging | Disease | Disease | 0.995 | 0.1208 | Gain of sheet | No reliable reference | 4 |
| 24 | 1000 & HGMD | R141P | 1 | Probably Damaging | 0 | Damaging | Disease | Disease | 0.992 | 0.0735 | Loss of stability | No reliable reference | 4 |
| 25 | 1000 & HGMD | H168R | 1 | Probably Damaging | 0 | Damaging | Disease | Disease | 0.995 | 0.2251 | Loss of catalytic residue at Q171 | No reliable reference | 4 |
| 26 | 1000 & HGMD | H168Q | 1 | Probably Damaging | 0 | Damaging | Disease | Disease | 0.998 | 0.3585 | Loss of catalytic residue at Y167 | No reliable reference | 4 |
| 27 | 1000 & HGMD | N199D | 1 | Probably Damaging | 0 | Damaging | Disease | Disease | 0.989 | 0.1208 | Gain of sheet | No reliable reference | 4 |
| 28 | 1000 & HGMD | N199S | 0.999 | Probably Damaging | 0 | Damaging | Disease | Disease | 0.988 | 0.2237 | Loss of loop | No reliable reference | 4 |
| 29 | Novel Mutation | N199H | 1 | Probably Damaging | 0 | Damaging | Disease | Disease | 0.945 | - | - | No reliable reference | 4 |
| 30 | 1000 & HGMD | D263N | 1 | Probably Damaging | 0 | Damaging | Disease | Disease | 0.989 | 0.1299 | Loss of helix | No reliable reference | 4 |
| 31 | 1000 & HGMD | C303Y | 0.995 | Probably Damaging | 0 | Damaging | Disease | Disease | 0.978 | 0.0519 | Gain of phosphorylation at C303 | No reliable reference | 4 |
| 32 | 1000 & HGMD | R330G | 0.996 | Probably Damaging | 0 | Damaging | Disease | Disease | 0.983 | 0.051 | Loss of stability | No reliable reference | 4 |

TABLE S2**:** Z-score, binding affinities, RMSDs and hydrogen bond interactions of native and mutant models.

| **Residue** | **ProSA-Web** | **Binding affinity (kcal/mol)** | **RMSD between PALO in native and PALO in mutant structures** | **Residue interact with**  **PALO at ligand binding pocket** | **Native residue interact with neighboring residue**  **Native residue (Atom): Neighboring residue (Atom)** | **Mutant residue interact with neighboring residue**  **Mutant residue (Atom): Neighboring residue (Atom)** |
| --- | --- | --- | --- | --- | --- | --- |
|  | **Z-score** |  |  |  |  |  |
| Native | -8.87 | -8.4 | 0.110 | S90, T91, R92, T93, R141, H168, N199, D263, S267, M268, L304, R330 | -NA- | -NA- |
| *Q171H | -8.90 | -8.4 | 0.110 | S90, T91, R92, T93, R141, H168, N199, D263, S267, M268, L304, R330 | Q171(N):H168(O)  Q171(NE2):D175(OD1)  Q171(NE2):E326(OE1)  Q171(O):A174(N)  Q171(O):D175(N)  Q171(OE1):R330(NH1) | H171(N):H168(O)  H171(O):A174(N)  H171(O):D175(N) |
| Q171E | -8.88 | -8.4 | 0.120 | S90, T91, R92, T93, R141, H168, N199, D263, S267, M268, L304, R330 |  | E171(N):H168(O)  E171(O):A174(N)  E171(O):D175(N)  E171(OE2):D175(OD2)  E171(OE1):R330(NH1) |
| *N199H | -8.86 | -7.8 | 1.737 | S90, T91, R92, T93, R141, H168, D263, S267, M268, L304, R330  Loss of interaction with: H199 | N199(ND2):S164(O)  N199(OD1):I200(N)  N199(O):S203(N)  N199(O):S203(OG) | H199(O):S203(N)  H199(O):S203(OG) |
| N199D | -8.86 | -8.1 | 0.205 | S90, T91, R92, T93, R141, H168, D199, D263, S267, M268, L304, R330 |  | D199(O):S203(N)  D199(O):S203(OG) |
| N199S | -8.90 | -8.3 | 0.553 | S90, T91, R92, T93, R141, H168, S199, D263, S267, M268, L304, R330  Form new interaction with: N198 |  | S199(O):S203(N)  S199(O):S203(OG) |
| R92P | -9.02 | -6.5 | 0.342 | S90, T91, R141, H168, N199, D263, S267, M268, L304, R330  Loss of interaction with: P92 and T93 | R92(O):S96(N)  R92(O):S96(OG) | P92(O):S96(N)  P92(O):S96(OG) |
| R92G | -8.93 | -7.9 | 0.119 | S90, T91, G92, T93, R141, H168, N199, D263, S267, M268, L304, R330 |  | G92(O):S96(N)  G92(O):S96(OG) |
| R92L | -8.84 | -8.1 | 0.237 | S90, T91, L92, T93, R141, H168, D263, S267, M268, L304, R330  Loss of interaction with: N199 |  | L92(O):S96(N)  L92(O):S96(OG) |
| R92Q | -8.86 | -8.1 | 0.152 | S90, T91, Q92, T93, R141, H168, N199, D263, S267, M268, L304, R330 |  | Q92(O):S96(N)  Q92(O):S96(OG) |
| H168R | -8.82 | -6.9 | 2.027 | S90, T91, R92, R141, D263, S267, M268, L304, R330  Loss of interaction with: R168, T93  N199, S267  Form new interaction with: P305 and K307 | H168(N):S203(OG)  H168(O):Q171(N) | R168(N):S203(OG)  R168(O):Q171(N) |
| H168Q | -8.87 | -7.6 | 0.511 | S90, T91, R92, T93, R141, Q168, N199, D263, S267, M268, L304, R330 |  | Q168(N):S203(OG)  Q168(O):Q171(N) |
| H168P | -8.92 | -8.1 | 0.171 | S90, T91, R92, T93, R141, N199, D263, S267, M268, L304, R330  Loss of interaction with: P168 |  | P168(N):S203(OG)  P168(O):Q171(N) |
| S90G | -8.88 | -8.3 | 0.148 | T91, R92, T93, R141, H168, N199, D263, S267, M268, L304, R330  Loss of interaction with: G90 | S90(OG):R94(N) | -NA- |
| S90N | -8.87 | -8.3 | 0.143 | T91, R92, T93, R141, H168, N199, D263, S267, M268, L304, R330  Loss of interaction with: N90 |  | -NA- |
| S90R | -8.80 | -8.3 | 0.171 | T91, R92, T93, R141, H168, N199, D263, S267, M268, L304, R330  Loss of interaction with: R90 |  | -NA- |
| T91I | -8.76 | -8.4 | 0.165 | S90, I91, R92, T93, R141, H168, N199, D263, S267, M268, L304 | L95(N):T91(O) | L95(N):I91(O) |
| T93A | -8.80 | -7.5 | 0.122 | S90, T91, Q92, A93, R141, H168, N199, D263, S267, M268, L304, R330 | T93(O):S96(OG)  T93(O):T97(N)  T93(O):T97(OG1)  T93(OG1):R141(NH2)  T93(OG1):R330(NH2) | A93(O):S96(OG)  A93(O):T97(N)  A93(O):T97(OG1) |
| L163P | -8.86 | -8.1 | 0.740 | S90, T91, R92, T93, R141, H168, N199, D263, S267, M268, R330  Loss of interaction with: L304 | L163(O):V142(N)  L163(N):N161(OD1) | P163(O):V142(N) |
| L304F | -8.92 | -8.4 | 0.130 | S90, T91, R92, T93, R141, H168, N199, D263, S267, M268, F304, R330 | L304(N):H302(ND1) | F304(N):H302(ND1) |
| R330G | -8.90 | -8.0 | 0.163 | S90, T91, R92, T93, R141, H168, N199, D263, S267, M268, L304  Loss of interaction with: G330 | R330(N):A327(O)  R330(NH1):Q171(OE1)  R330(NH2): T93(OG1)  R330(O):T333(OG1)  R330(O):I334(N) | G330(N):A327(O)  G330(O):T333(OG1)  G330(O):I334(N) |
| R141G | -9.05 | -7.6 | 0.110 | S90, T91, R92, T93, H168, N199, D263, S267, M268, L304, R330  Loss of interaction with: G141 | R141(O):E87(N)  R141(N):I85(O)  R141(NE):G162(O)  R141(NH1):R89(O)  R141(NH2):T93(OG1)  R141(NH2):G162(O) | G141(O):E87(N)  G141(N):I85(O) |
| R141L | -8.90 | -7.6 | 0.176 | S90, T91, R92, T93, H168, N199, D263, S267, M268, L304, R330  Loss of interaction with: L141 |  | L141(O):E87(N)  L141(N):I85(O) |
| R141P | -9.10 | -7.6 | 0.140 | S90, T91, R92, T93, H168, N199, D263, S267, M268, L304, R330  Loss of interaction with: P141 |  | P141(O):E87(N) |
| R141Q | -8.83 | -7.7 | 0.117 | S90, T91, R92, T93, H168, N199, D263, S267, M268, L304, R330  Loss of interaction with: Q141 |  | Q141(O):E87(N)  Q141(N):I85(O)  Q141(NE2):R89(O) |
| D263N | -8.89 | -7.9 | 1.738 | S90, T91, R92, T93, R141, H168, N263, M268, L304, R330  Loss of interaction with: N199  Form new interaction with: S267 | D263(OD2):N198(ND2)  D263(O):T262(OG1)  D263(N):T262(OG1)  D263(OD1):T264(N) | N263(O):T262(OG1) N263(N):T262(OG1)  N263(OD1):T264(N)  N263(OD1):C303(SG) |
| D263G | -8.93 | -8.0 | 0.131 | S90, T91, R92, T93, R141, H168, N199, S267, M268, L304, R330  Loss of interaction with: G263 |  | G263(O):T262(OG1)  G263(N):T262(OG1)  G263(N):G263(O) |
| D263Y | -8.94 | -8.2 | 0.141 | S90, T91, R92, T93, R141, H168, N199, S267, M268, L304, R330  Loss of interaction with: Y263 |  | Y263(OH ):N198(N)  Y263(OH):N198(ND2)  Y263(O):T262(OG1)  Y263(N):T262(OG1)  Y263(OH):D196(O) |
| S267R | -8.88 | -8.2 | 0.731 | S90, T91, R92, T93, R141, H168, N199, D263, M268, L304, R330  Loss of interaction with: R267 | R267(O):R270(N) | R267(O):R270(N) |
| M268T | -8.96 | -8.4 | 0.168 | S90, T91, R92, T93, R141, H168, N199, D263, S267, T268, L304, R330 | -NA- | -NA- |
| C303R | -8.79 | -8.0 | 1.380 | S90, T91, R92, T93, R141, N199, D263, S267, M268, L304  Loss of interaction with: H168 and R330  Form new interaction with:D263 | -NA- | R303(NH1):H168(ND1)  R303(NH2):H168(ND1) |
| C303Y | -8.92 | -8.0 | 1.255 | S90, T91, R92, T93, R141, H168, D263, M268, L304, R330  Loss of interaction with: N199  Form new interaction with: S267 |  | Y303(OH):D175(OD2) |
| C303G | -8.90 | -8.3 | 0.288 | S90, T91, R92, T93, R141, H168, N199, D263, S267, M268, L304, R330 |  | -NA- |

*Novel mutation reported in this study.
